# Supplementary material for: An Eco-Friendly Adsorbent Based on Bacterial Cellulose and Vermiculite Composite for Efficient Removal of Methylene Blue and Sulfanilamide
Source: Polymers (Basel). 2023 May 17;15(10):2342. doi: 10.3390/polym15102342 (PMC10222638; doi:10.3390/polym15102342)
Supplement: Supplementary file 1 [file polymers-15-02342-s001.zip › polymers-2326545-supplementary.pdf]

polymers

Supplementary Information

An Eco-Friendly Adsorbent Based on Bacterial Cellulose and Vermiculite Composite for Efficient Removal of Methylene Blue and Sulfanilamide

Xiuzhi Bai [1], Zhongxiang Liu [1], Pengfei Liu [1,2,*], Yijun Zhang [1], Linfeng Hu [1,3] and Tongchao Su [4]

[1] School of Chemistry and Chemical Engineering, Henan Institute of Science and Technology, Xinxiang 453003, China; amibai@126.com (X.B.); liuzhongxiang0903@126.com (Z.L.); zhangyijun@hist.edu.cn (Y.Z.)
[2] School of Chemistry and Chemical Engineering, Hunan University of Science and Technology, Xiangtan 411201, China
[3] Experiment and Test Center, Henan Institute of Science and Technology, Xinxiang 453003, China
[4] School of Food Science, Henan Institute of Science and Technology, Xinxiang 453003, China; sutongchao66@163.com
* Correspondence: lpf8856@163.com

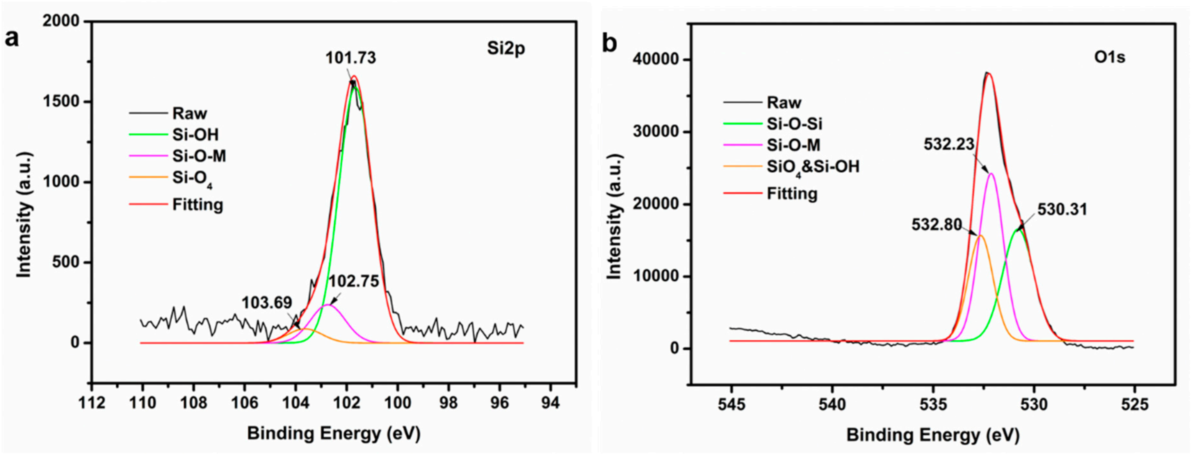

Figure S1. The high-resolution XPS spectra of Si2p (a) and O1s (b).

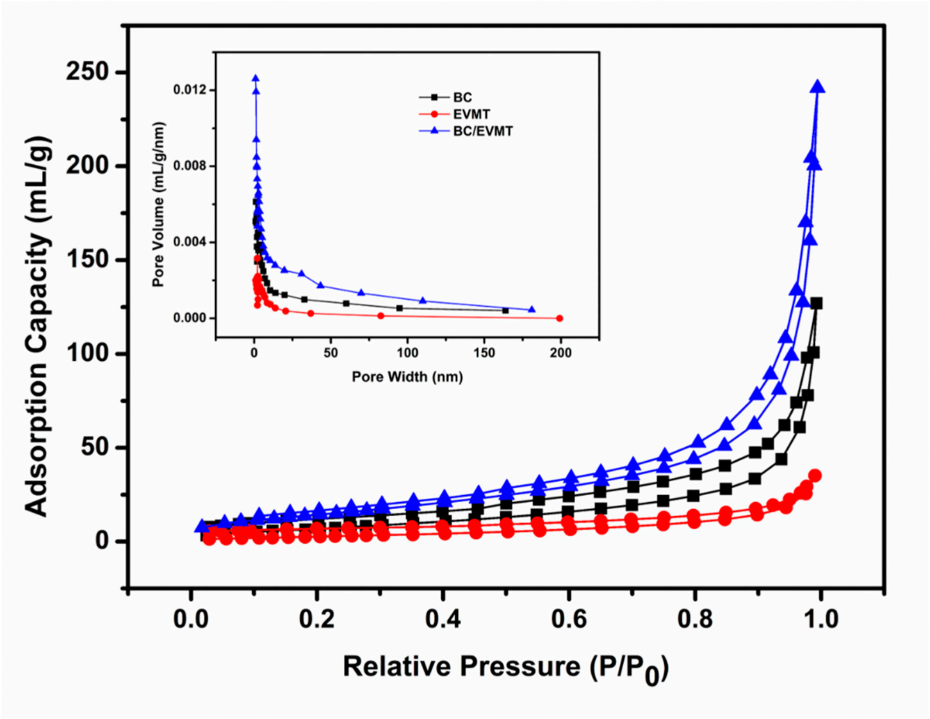

Figure S2. The nitrogen adsorption-desorption isotherms of EVMT, BC, and BC/EVMT by Brunauer-Emmett-Teller (BET) method.

Table S1. BET data of EVMT, BC, and BC/EVMT.

Samples	BET surface area (m^2 g^{-1})	BJH Adsorption cumulative volume of pores (mL g^{-1})	Adsorption average pore width (nm)
BC	28.67	0.1964	12.47
EVMT	11.50	0.0543	10.43

BC/EVMT	55.70	0.3739	17.48

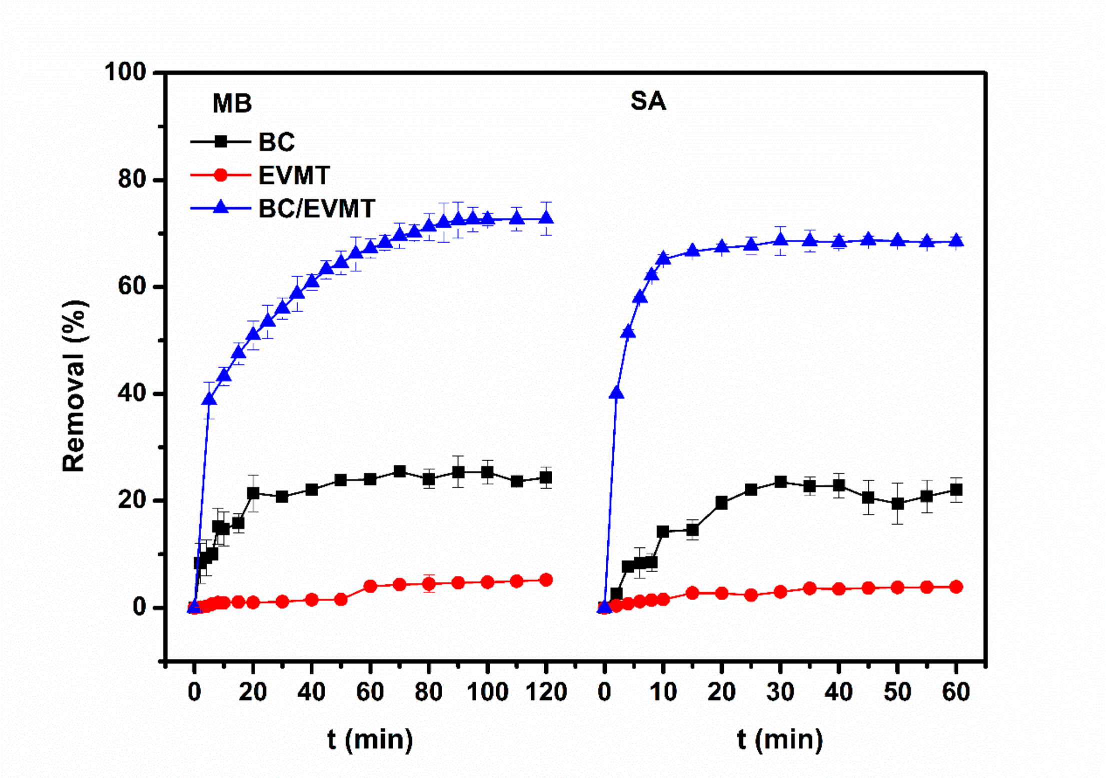

Figure S3. Removal efficiency of BC, EVMT, and BC/EVMT for MB and SA (temperature: 30 °C, pH_{MB}: 8, pH_{SA}: 2, adsorbent dosage: 20 mg, $[MB]_0$: 60 mg/L, $[SA]_0$: 60 mg/L).

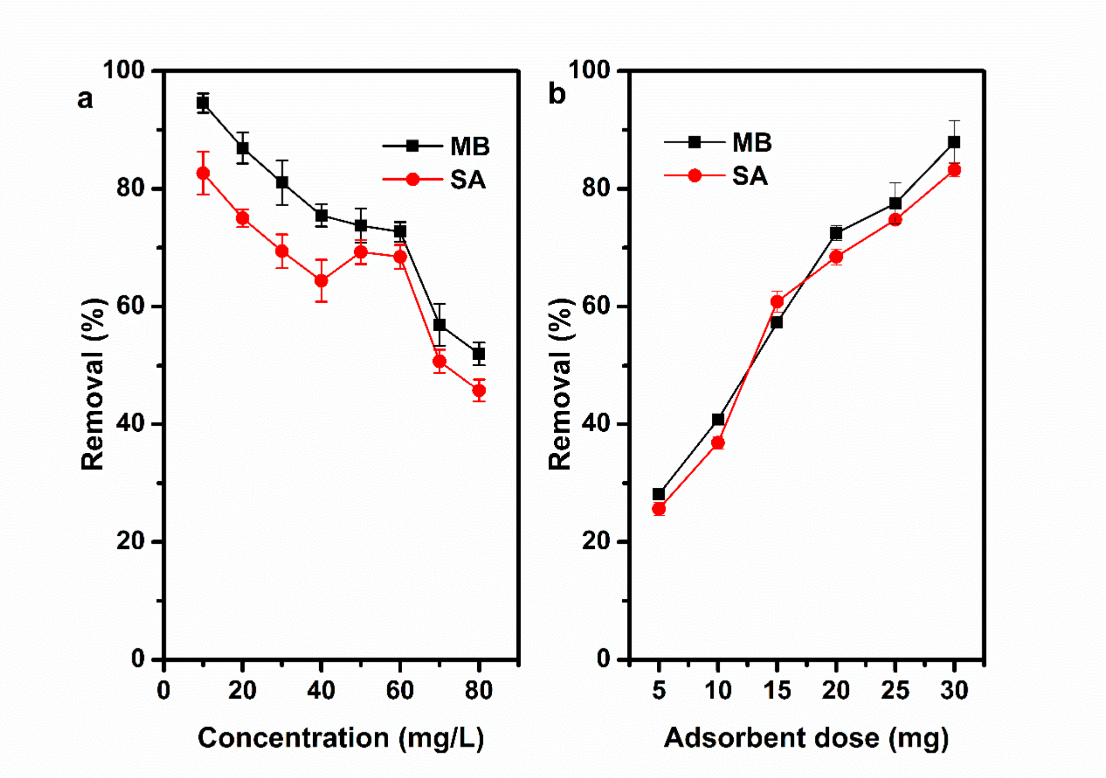

Figure S4. Effect of various experimental parameters on the adsorption of MB and SA by BC/EVMT: (a) MB and SA concentration and (b) adsorbent dosage. (temperature: 30 °C, pH_{MB}: 8, pH_{SA}: 2, adsorbent dosage: 20 mg, $[MB]_0$: 60 mg/L, $[SA]_0$: 60 mg/L, t: 120 min).
